# Antimicrobial resistance varies with warming in active layer soil and permafrost

**DOI:** 10.1038/s41598-026-46295-2

**Published:** 2026-04-14

**Authors:** Quincy Faber, Christopher C. M. Baker, Jaimie R. West, Stacey J. Doherty, Jessica G. Ernakovich, Robyn A. Barbato

**Affiliations:** 1 Engineer Research Development Center, Cold Regions Research and Engineering Laboratory, Biogeochemical Sciences Branch, U.S. Army Corps of Engineers, Hanover, NH USA; 2 University of New Hampshire, Durham, NH USA

**Keywords:** Antimicrobial resistance, Permafrost, Thaw, Metagenomics, Soil microbiome, Temperature stress, Ecology, Ecology, Environmental sciences, Microbiology

## Abstract

Although antimicrobial resistance is a contemporary public health concern, antimicrobial resistance genes (ARGs) have existed long before human use of antimicrobials, and recent attention has focused on whether permafrost thaw could release ARGs as the resistome shifts. We present a metagenomic analysis of permafrost samples from four sites in Alaska and Sweden, thawed under laboratory conditions. We used ABRicate, an alignment-based tool, and DeepARG, a deep learning tool, to identify ARGs, assessed their abundances under experimental thaw, measured taxonomic shifts, and examined metagenome-assembled genomes (MAGs) carrying ARGs. ARG abundance varied with depth, with some permafrost containing more ARGs than the seasonally thawed active layer. ARG abundance increased with soil carbon and decreased with pH across sites, suggesting site-specific influences. The majority of 164 high-quality MAGs contained ARGs, including 80 out of 105 species identified. This included bacteria from nine phyla, demonstrating widespread distribution across microbial taxa. Laboratory thaw experiments revealed that ARG abundances did not change significantly in two of the sites, but declined with thaw in the remaining two sites. Together, these findings demonstrate that ARGs are consistently present in permafrost microbiomes across multiple sites, but relative abundances generally do not increase during thaw. While ARGs that persist may pose potential risks, our results suggest that permafrost thaw may not substantially elevate environmental or public health risks.

**Supplementary Information:**

The online version contains supplementary material available at 10.1038/s41598-026-46295-2.

## Introduction

Antimicrobial resistance genes (ARGs) are genes found in microorganisms, primarily bacteria, that confer resistance to antimicrobials. The soil microbiome serves as a reservoir of abundant and diverse ARGs that improve bacterial survival in competitive soil environments^1,2 ^and are thought to play a role in cell signaling^3^. Although anthropogenic activities such as animal husbandry and agriculture increase ARG levels in soils^4,5^, antimicrobial resistance (AMR) predates human antimicrobial use^6,7^. The soil resistome is known to harbor novel ARGs that are often absent from clinical databases yet share similarities with clinically relevant resistance genes^2,6,8,9^. Understanding the evolution of AMR in the environment and its transmission is necessary for mitigating future public health risks^10^.

As permafrost regions rapidly warm^11^, thawing may mobilize ancient resistance genes into modern ecosystems^12^. Bacterial strains resistant to aminoglycosides, beta lactamases, fosfomycin, rifamycin, and tetracyclines have been isolated from cold regions soils and permafrost^13–19^. Interestingly, two studies comparing temperate soil strains to permafrost strains reported conflicting trends regarding ARG enrichment: one study^17^ found comparable levels of AMR in permafrost and modern isolates, whereas another study^18^ reported that genomes of modern isolates are enriched in ARGs relative to permafrost strains. ARGs have been well-documented in metagenomes of soil and permafrost from polar regions including Antarctica^20–23^, Svalbard^24^, Siberia^25,26^, northern Sweden^27^, and Alaska^28^, and in high altitude cold regions such as the Tibetan Plateau^29,30^. Soil depth has been shown to impact ARG distribution, with higher abundances typically observed in seasonally thawed active layer soils closest to the surface compared to deeper permafrost^28^. In addition, permafrost type and thaw stage also matter: greater ARG abundances have been observed in more thawed bog permafrost compared to intact palsa permafrost^27^.

While horizontal gene transfer is a major driver of ARG mobility in clinical settings, the ARG composition in soils is largely shaped by microbial phylogeny and environmental conditions^3,31^. Environmental factors that influence microbial community composition—such as nutrients, temperature, and freeze-thaw cycles—may therefore also affect ARG distribution^31,32^. Permafrost soils are naturally enriched in heavy metals due to chemical weathering of bedrock and anaerobic microbial processes that increase metal mobilization^33^. Because genes conferring resistance to heavy metals are often co-located with ARGs or share cellular pathways^34^, this accumulation may indirectly select for AMR in permafrost microbial communities^35^. AMR may additionally be favored in environments with strong microbial competition, as suggested by studies showing higher ARG abundance in young soils with limited organic carbon^36^.

Few studies have investigated the impact of permafrost thaw on ARG classes and abundances. A study in Abisko, Sweden found that despite differences in microbial community properties across a natural permafrost thaw gradient, the overall abundance of ARGs did not differ significantly between intact and thawed permafrost^37^. Another study observed increased abundances of ARGs with increased thaw across a natural thaw gradient, but these changes were driven by differences in phylogeny^32^. Studies in other soil environments suggest potential changes that may also be relevant for thawing permafrost. For example, freeze–thaw cycling has been shown to increase ARG abundances in soils^38^ and higher environmental temperatures are associated with greater prevalence of AMR across a broad range of pathogens^39^. While several studies have documented changes in microbial community composition during both natural and laboratory stimulated permafrost thaw^40–42^, the effect of permafrost thaw on relative ARG abundances remains underexplored.

Several methods exist for identifying ARGs in metagenomes. Alignment-based tools, such as ABRicate^43^ and ARG-ANNOT^44^ Alignment Search Tool (BLAST)^45^ to compare sequences against reference databases. More recently, machine learning-based tools have been developed, including DeepARG^46^ and fARGene^47^. DeepARG trains its models using BLAST alignments^46^ and achieves high precision (few false positives) as well as strong recall (few false negatives). Importantly, DeepARG can identify novel ARGs within both common and rare classes because it is trained on databases that include environmental and uncultured microbial sequences, such as Uniprot^48^, giving it broader coverage than clinically focused ARG databases such as the Comprehensive Antibiotic Resistance Database^49^.

In this study, we aimed to characterize the permafrost resistome and its response following thaw, hypothesizing that AMR increases with thaw, and is linked to thaw-driven changes in microbial community composition. Permafrost cores from several Arctic and subarctic sites were incubated under thaw conditions spanning 4 to 15 °C for 16 d to 193 d. We then explored how the presence of ARGs, assessed with both alignment-based and deep learning-based tools, and microbial community composition respond to thaw across diverse permafrost environments.

## Methods

### Sample collection and laboratory incubations

**Fig. 1:**
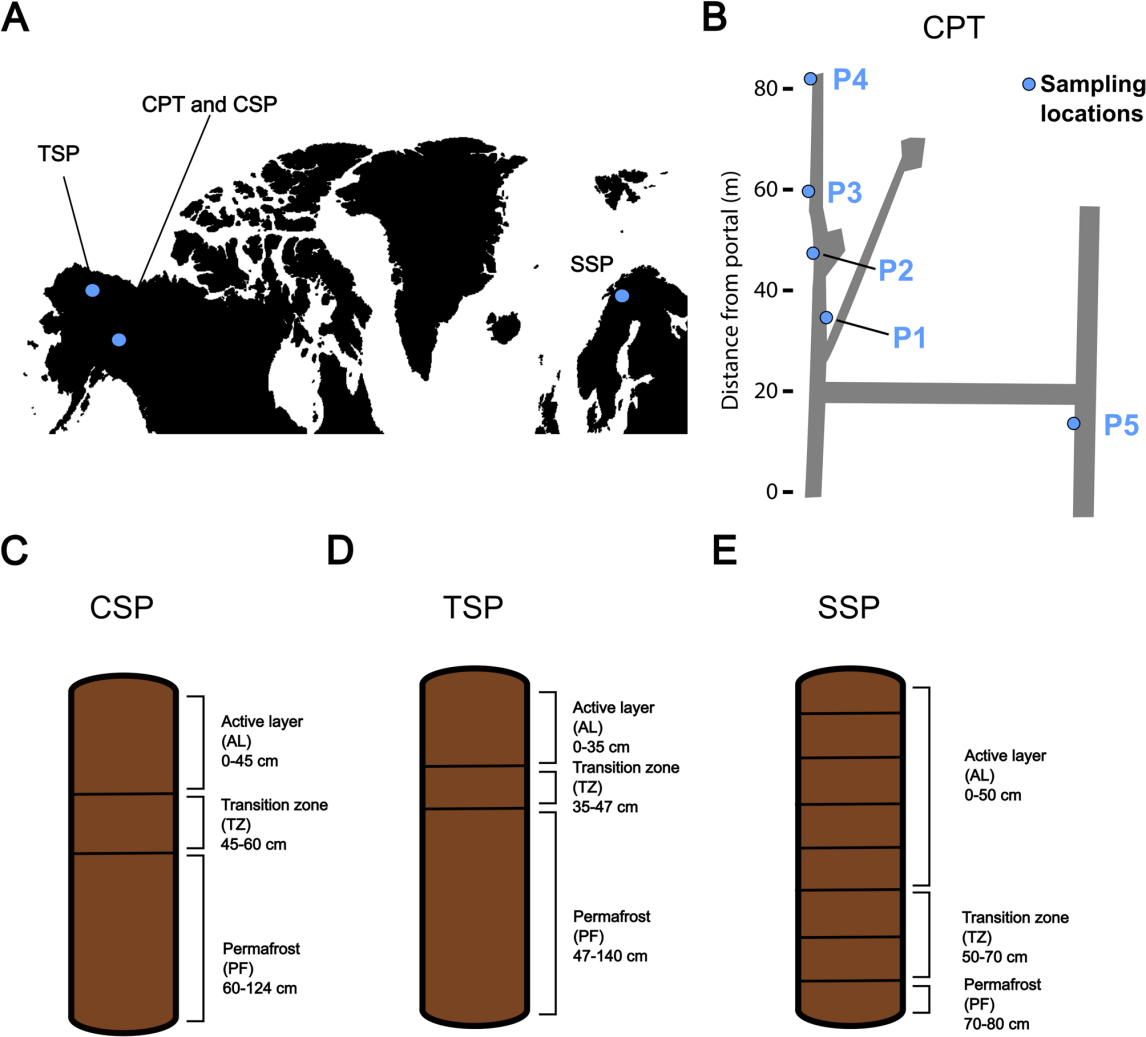
Sampling locations and depths of soil profiles. (**A**) Map with sample sites marked in blue, generated with the maps package in R^50^. (**B**) Map of the CRREL Permafrost Tunnel (CPT) in Fox, Alaska, USA with sampling locations indicated in blue. (adapted from^51^).** (C**) Core subsampling of soil profiles above the CRREL permafrost tunnel (CSP). (**D**) Core subsampling of soil profiles at thermokarst sites near Toolik Lake (TSP). (**E**) Core subsampling representing 4 cores collected from Abisko, Sweden soil profiles (SSP).

We used data from three previously published laboratory incubation studies encompassing permafrost from Alaska^51^, permafrost soil profiles from Alaska^52^, and permafrost soil profiles from Sweden^53^. Collection and incubation of permafrost from the US Army Cold Regions Research and Engineering Laboratory Permafrost Tunnel Research Facility (CPT) in Fox, Alaska, USA (64.9528°N, 147.6178°W; Fig. 1A, B) is described in detail in Barbato et al.^51^. The collection and incubation of Alaska soil profiles (Fig. 1C, D) from above the CRREL Permafrost Tunnel Research Facility in Fox, AK (CSP; 64.9507°N, 147.6200°W) and a thermokarst site close to the Toolik Lake Field Station (TSP; 68.649265°N, 149.509637°W) is described in Doherty et al.^52^. Sample collection and laboratory incubation of soil profiles from the Storflaket Mire in Abisko, Sweden (SSP; 68.3466°N, 18.9711°E; Fig. 1E) is described in Doherty et al.^53^. The soils and permafrost analyzed in this study span continuous, discontinuous, and sporadic permafrost zones and include both high organic matter peat and ice-rich mineral soils (Table S1). The pH of all samples was measured, organic matter content was determined for the CPT dataset, and total C and N were quantified for the CSP, TSP, and SSP datasets; details of these analyses are provided in the supplemental methods. A detailed list of all samples is included in Table S2.

### DNA extraction and shotgun sequencing

**Table 1:** Summary of datasets and incubations.

Sampling locations	Sampling and incubation dates	Storage conditions	Incubation length (days)	Incubation temperatures	DNA extraction and cleanup	Library preparation method	Sequencing platform
CRREL Permafrost Tunnel (CPT)	July 2017; February 2018	Stored at -20 °C, then transferred to -80 °C; processed at -10 °C	16	-3 °C for 4 d, 0 °C for 6 d, 3 °C for 3.5 d, and 6 °C for 2.5 d (sequential)	DNeasy PowerLyzer PowerSoil kit (Qiagen)	Wafergen’s PrepX Complete ILMN DNA library Kit	Illumina HiSeq 2500 (2 × 100 bp)
CRREL Soil Profile (CSP)	March 2023; October 2023	Stored at -80 °C; held at 4 °C for 2 days prior to incubation	100	10 °C	DNeasy PowerSoil Pro kit (Qiagen) with QIAcube automated DNA extractor	Illumina TruSeq DNA Library Prep Kit v2	Illumina NextSeq 2000 (2 × 150 bp)
Toolik Soil Profile (TSP)	October 2019; October 2023	Stored at -10 °C; held at 4 °C for 2 days prior to incubation	100	10 °C	DNeasy PowerSoil Pro kit (Qiagen) with QIAcube automated DNA extractor	Illumina TruSeq DNA Library Prep Kit v2	Illumina NextSeq 2000 (2 × 150 bp)
Sweden Soil Profile (SSP)	June 2019; July 2019	Stored at -20 °C; incubation samples thawed for 12 days, then stored at 4 °C for 3 days	193	4,15 °C	DNeasy PowerSoil kit (Qiagen), followed by MoBioPowerClean kit (MoBio)	Illumina TruSeq DNA Library Prep Kit v2	Illumina NextSeq 2000 (2 × 150 bp)

Incubations conditions, DNA extractions, library extractions, and sequencing platforms are described in Table 1. Pre-incubation DNA samples were collected from CPT and CSP samples following thaw for 2 d at 4 °C and from frozen SSP samples. CPT samples were collected following incubation at -3 °C, 0 °C, and 6 °C. With the exception of SSP, replicates were derived from the same homogenized soil sample. SSP replicates were collected from four distinct permafrost cores. DNA was extracted following the manufacturer’s protocols using 0.25 g of soil. For SSP samples, DNA was extracted in triplicate and pooled onto one spin filter. Shotgun metagenomic library preparation and sequencing was completed at the Next Generation Sequencing Core at the Argonne National Laboratory.

### Read processing and taxonomic analysis

BBDuk (bbtools v.37.62^54^) was used to remove adapters from the 3’ ends of reads, trim bases falling below a Phred score of 10, remove reads shorter than 50 bp, and eliminate any residual PhiX sequences. FastUniq (v.1.1^55^) was used to deduplicate remaining reads. Taxonomic profiles were generated for each sample based on reads using MetaPhlAn (v.4.1.1^56^) with the mpa_vJun23_CHOCOPhlAnSGB_202403 database. We calculated Bray-Curtis dissimilarities using the calculate_diversity.R script from the MetaPhlAn repository, and plotted results in R with the help of the vegan package^57^.

### Assembly and gene prediction

Metagenomic reads were assembled *de novo* for each sample using MEGAHIT (v1.2.9^58^) with default parameters (k-mer sizes 21–141 in steps of 10, minimum contig length 200 bp, bubble merging and pruning enabled, preset meta-large). Following assembly, Prodigal (v2.6.3^59^) was used to identify and predict protein-coding genes on the assembled contigs using default settings, which applies a self-trained model with a minimum gene length of 90 bp and the standard bacterial/archaeal translation table (code 11).

### Metagenome-assembled genomes

Binning was performed using MaxBin (v2.2.7^60^) with default settings, using assembled contigs as the primary input and quality-controlled reads to provide coverage information. Checkm2 (v1.0.1^61^) was used to estimate completeness and contamination. Bins with > 90% completeness and < 5% contamination were considered metagenome-assembled genomes (MAGs). Taxonomic classification of MAGs was done using GTDB-Tk (v2.4.1^62^) using the GTDB R226 database.

### Antimicrobial-resistance gene analysis

ARGs were identified in predicted genes and MAGs using the deep learning tool DeepARG (v2^46^). Default settings were used, including minimum probability cutoff (0.8), alignment e-value (1e-10), and ARG alignment identity (50). CD-HIT (v4.8.1^63^) was run with a 100% sequence identity threshold and word size of 5 to remove duplicate genes identified by more than one database. ARGs were also predicted using ABRicate (v1.0.1^43^; https://github.com/tseemann/abricate) with default settings (75% identity cutoff, 80% coverage cutoff, 1e-6 e-value threshold) with the following databases: NCBI AMRFinderPlus (v4.0.3^64^), CARD (v4.0.1^49^), Resfinder^65^, ARG-ANNOT (v6^44^), and MEGARES (v3.0.0^66^). HAMRonization was used to summarize reports from ABRicate and DeepARG (v1.1.9^67^). Paired-end reads were mapped to ARG reference sequences for each dataset using Bowtie2 (v2.5.4^68^) with default settings. Read abundance for each gene was normalized to Reads Per Kilobase per Million mapped reads (RPKM^69^ ) using Eq. 1 to allow for comparison between samples and datasets:

1 $$\:RPKM=\:\frac{Number\:of\:Mapped\:Reads}{Gene\:Length\:in\:kb\:\times\:\:\frac{Total\:Reads}{\mathrm{1,000,000}}}$$

### Statistical analysis

Differences in the relative abundance of ARGs across sites, across depths within each site, and between time points at each site were assessed using statistical tests appropriate for the data distribution. For each comparison, residuals were first tested for normality using the Shapiro-Wilk test (base R) and homogeneity of variances using Levene’s test (car package in R ^70^). When parametric assumptions were met, one-way ANOVA was used, followed by Tukey’s HSD post hoc tests to identify specific group differences. When parametric assumptions were not met, non-parametric tests were applied: the Kruskal-Wallis test for overall group comparisons^71^ , followed by pairwise Wilcoxon rank-sum tests^72^ .

Ordination was performed using non-metric multidimensional scaling (NMDS) with the metaMDS function and Principal Coordinates Analysis (PCoA) via classical metric multidimensional scaling (cmdscale) based on Bray–Curtis dissimilarities in R ^57,73^. Permutational multivariate analysis of variance (PERMANOVA) was performed using the adonis2 function from the vegan package in R to test for differences in community composition. For baseline comparisons at timepoint 0 (t_0_), effects of site and depth were evaluated separately. To assess the effect of thaw stage across the full dataset, PERMANOVA was run with site and depth included as covariates to account for spatial variation. Differences in relative abundance of taxonomic groups at the phylum and class levels by Time and Site were tested using ANOVA, with p-values adjusted using the Benjamini-Hochberg method^73,74^ . Significance was defined as *P* < 0.05, and all statistical analyses were performed in R.

## Results

Antimicrobial resistance genes (ARGs) were assessed in four permafrost metagenome datasets before and after laboratory incubations. Sequencing depth varied between sites, but on average, over 10 million paired-end reads were sequenced from each sample (Table S3). DeepARG recovered hundreds to thousands of ARGs in each site spanning 15 to 20 resistance classes that were consistent across all sites, whereas ABRicate identified only a small number of ARGs per site and typically captured just 3 to 4 resistance classes. Full details of ARGs identified are provided in Table S4 for DeepARG and Table S5 for ABRicate.

### ARG abundances across sites

**Fig. 2:**
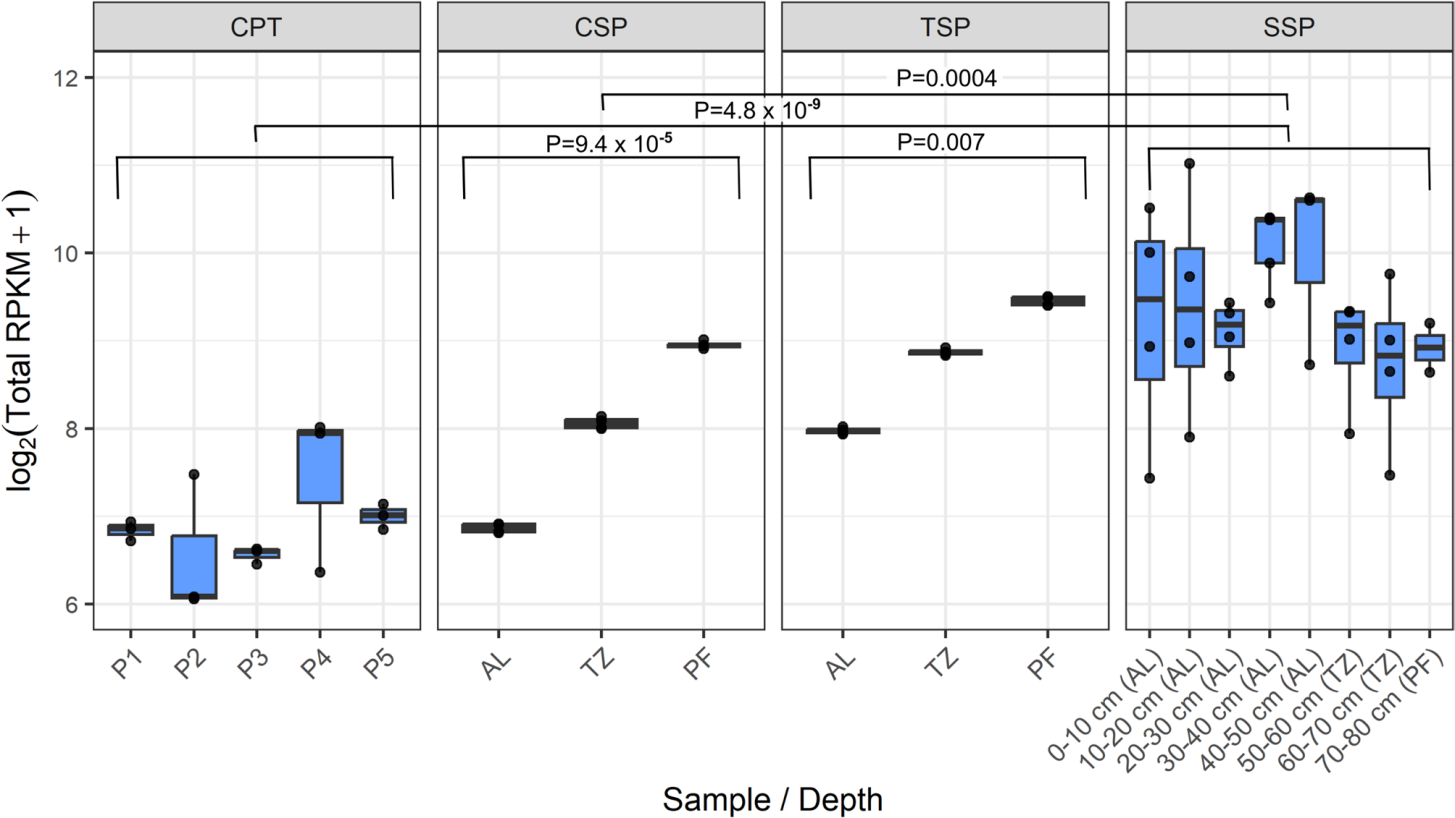
Relative abundance of antimicrobial resistance genes across sample sites prior to incubation, as estimated by DeepARG. RPKM = Reads Per Kilobase of gene per Million mapped reads. RPKM values are displayed as log_2_(RPKM + 1). AL= active layer, TZ= transition zone, PF= permafrost. Error bars indicate standard deviation.

Relative abundance of ARGs was calculated as Reads Per Kilobase of gene per Million mapped reads (RPKM) by mapping metagenomic reads to resistance genes (Fig. 2). DeepARG analysis showed that SSP profiles had the greatest abundance (775.09 ± 484 RPKM), followed by TSP (472 ± 192 RPKM), CSP (292 ± 163 RPKM), and CPT (127 ± 58.3 RPKM). Differences across sites were highly significant for DeepARG results (Kruskal-Wallis, *P* = 0.0003) with pairwise comparisons showing that SSP had higher relative abundances than CSP (Wilcoxon rank-sum test, BH-corrected; *P* = 0.0004) and CPT (Wilcoxon rank-sum test, BH-corrected; *P* = 4.8 × 10⁻⁹). ABRicate results were similar but an order of magnitude lower, ranging from 0 to 80 RPKM (Fig. S1). SSP samples contained the highest relative abundance of ARGs, but were not statistically different from CSP and TSP.

Soil properties varied significantly between each site (Kruskal-Wallis, *P* < 0.001), although total C was measured through loss on ignition for CPT (see supplemental methods) and total N was only available for the CSP, TSP, and SSP datasets (Table S6). SSP and TSP had the highest C % at 37.1 and 15.9%, respectively. Soil properties differ between AL and PF, with pH, C%, and N% all higher at AL (pH *P* = 0.0119, C% *P* = 0.00012, N% *P* = 0.0033). ARG abundance, as detected by both DeepARG and ABRicate, was negatively correlated with soil pH, whereas ARG abundance was positively correlated with soil C content. Using DeepARG, correlations were moderate (pH: ρ = -0.39, *P* = 0.001; C %: ρ = 0.56, *P* = 1.1 × 10^− 6^; Fig. S2B, D) and with ABRicate, correlations were weak (pH: ρ = -0.29, *P* = 0.014; C %: ρ = 0.28, *P* = 0.02; Fig. S2A, C). Soil N content was also positively correlated to ARG abundance, but only with DeepARG results (ρ = 0.32, *P* = 0.018).

In the CPT samples, there were no significant differences in ARG abundance across sampling locations (one-way ANOVA, *n* = 15, DeepARG: *P* = 0.22; ABRicate: *P* = 0.52; Fig. 2, Fig. S22). For both Alaska soil profile sites, relative abundances of ARGs varied by depth. DeepARG results showed significant differences in ARG abundances by depth (CSP: one-way ANOVA, *n* = 12, *P* = 9.38 × 10⁻⁵ ; TSP: Kruskal-Wallis, *n* = 12, *P* = 0.007). Pairwise comparisons indicated that all three depths differed from each other. For CSP, Tukey’s HSD showed PF > AL (*P* = 9.55 × 10⁻⁵) and PF > TZ (*P* = 0.002), while TZ > AL was not significant (*P* = 0.398). For TSP, pairwise Wilcoxon tests with BH correction confirmed PF > TZ (*P* = 0.00047), PF > AL (*P* = 0.00062), and TZ > AL (*P* = 0.00047). ABRicate results also showed significant differences by depth (one-way ANOVA, *n* = 12; CSP: *P* = 0.006; TSP: *P* = 0.002; Fig. S1). Pairwise comparisons indicated that permafrost (PF) had higher relative abundances of ARGs than active layer (AL; CSP: PF > AL, *P* = 0.006; TSP: PF > AL, *P* = 0.002) and transition zone (TZ; CSP: PF > TZ, *P* = 0.027; TSP: PF > TZ, *P* = 0.013), whereas AL and TZ were not significantly different (CSP: *P* = 0.61; TSP: *P* = 0.45). In SSP samples, neither DeepARG nor ABRicate detected significant differences in ARG abundance across depth (DeepARG: one-way ANOVA *P* = 0.23; *n* = 30; ABRicate: Kruskal-Wallis, *P* = 0.28; Fig. 2, Fig. S1).

### Inconsistent shifts in ARG abundances with thaw

**Fig. 3:**
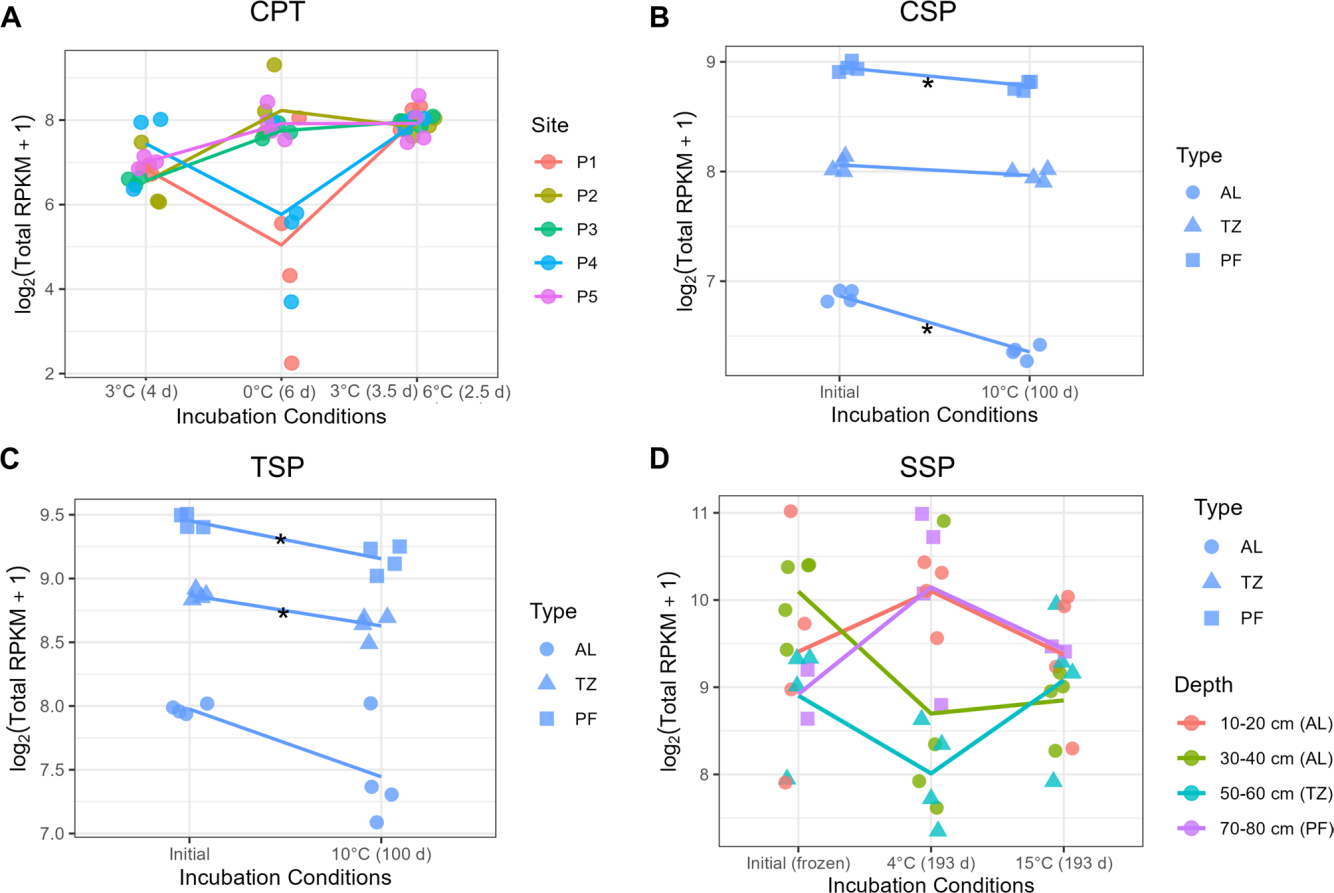
Relative abundance of antimicrobial resistance genes during laboratory thaw experiments, as estimated by DeepARG. Data are shown for CPT (**A**), CSP (**B**), TSP (**C**), and SSP (**D**) datasets. RPKM = Reads Per Kilobase of gene per Million mapped reads. RPKM values are displayed as log_2_(RPKM + 1). For CPT, CSP, and TSP, lines connect time points to show changes in ARG abundance. * denotes significant difference (*P* < 0.05) between pre and post thaw abundances. AL= active layer, TZ= transition zone, PF= permafrost.

Laboratory thaw experiments revealed variable changes in ARG abundance over time across datasets and depths (Fig. 3, Fig. S3). There was a significant effect of timepoint in the DeepARG results at several sampling locations (Kruskal-Wallis, P2: *P* = 0.045, P3: *P* = 0.016, P5: *P* = 0.050), but pairwise Wilcoxon tests with multiple testing correction did not reveal significant differences between specific time points (*P* > 0.05). However, ABRicate detected no significant changes in ARG abundance between initial (following − 3 °C incubation), intermediate (following 0 °C incubation), and final (following 3 °C and 6 °C incubation) time points when separated by sampling location (Kruskal-Wallis, *P* > 0.05; Fig. S3A). For CSP samples, ARG relative abundances decreased following incubation at 10 °C, with significant differences observed in AL (DeepARG: ANOVA, *P* = 1.4 × 10⁻⁵; ABRicate: Kruskal-Wallis, *P* = 0.021), TZ (one-way ANOVA; DeepARG: *P* = 0.06, not significant; ABRicate: *P* = 0.0002), and PF (one-way ANOVA; DeepARG: *P* = 0.0015; ABRicate: *P* = 0.020; Fig. 3B, Fig. S3B). For TSP samples, ARG relative abundances also decreased following incubation at 10 °C, with significant differences observed in permafrost (DeepARG: *P* = 0.002; ABRicate: one-way ANOVA, *P* = 0.021) and in the active layer and transition zone only in DeepARG (AL: *P* = 0.040; TZ: *P* = 0.002), while ABRicate showed no significant changes in AL or TZ (Fig. 3C, Fig. S3C). For SSP samples, DeepARG showed no significant changes in ARG abundance with temperature treatment (frozen, 4 °C, or 15 °C) when comparing different depths for SSP samples (one-way ANOVA; *P* > 0.05; Fig. 3D). However, ABRicate showed significant changes in ARG abundance with thaw for several depths (one-way ANOVA; 10–20 cm *P* = 0.039, 70–80 cm *P* = 0.005; Fig. S3D). Pairwise comparisons indicated that at 10–20 cm depth, relative abundances were higher following incubation at 15 °C compared to 4 °C (Tukey post-hoc, *P* = 0.025). At 70–80 cm depth, relative abundances were also higher at 15 °C compared to 4 °C (*P* = 0.007), and 4 °C samples were higher than frozen samples (*P* = 0.014).

### Broad diversity of ARG classes identified across sites

The relative abundances of ARG classes were assessed for t0 samples to characterize resistance gene distributions prior to thaw-induced shifts (Table S7; Figures S4 to S8). DeepARG detected a wide range of ARG classes at relatively high abundances across datasets, whereas ABRicate identified fewer classes and generally lower levels. A comparison of the beta-lactam ARG class, one of the most abundant classes, revealed a positive correlation between the two methods (Spearman’s ρ = 0.49, *P* = 3.95 × 10^− 8^). In the CPT samples, DeepARG showed multidrug and glycopeptide as the most abundant classes (49.9 and 33.4 RPKM). In CSP and SSP samples, DeepARG identified multidrug resistance genes as most prevalent, 80.69 RPKM and 159.9 RPKM, respectively. In TSP samples, DeepARG showed glycopeptide as the most abundant (56.2 RPKM). In the intact permafrost from CPT samples, neither DeepARG nor ABRicate detected significant differences in ARG class distribution between sampling locations (Kruskal-Wallis test, *P* > 0.05). Depth-related differences were observed in the recently thawed CSP samples, with DeepARG identifying significant differences for all classes except tetracenomycin and nucleoside (Kruskal-Wallis, *P* < 0.001) and ABRicate showing significant variation in rifamycin, multidrug, and polymyxin resistance across the active layer, permafrost, and transition zone. Generally, permafrost contained higher relative abundance of resistance genes compared to TZ and AL, except for Fosfomycin, which was more abundant in TZ (pairwise Wilcoxon tests, BH-adjusted, *P* < 0.05). In recently thawed TSP samples, ARG abundances varied significantly with depth. DeepARG identified depth-dependent differences in all classes except tetracenomycin, while ABRicate detected pairwise differences for multidrug and amphenicol resistance (Kruskal–Wallis, *P* < 0.05). In SSP samples, no significant depth-related differences in ARG classes were found by either DeepARG or ABRicate (Kruskal-Wallis, *P* > 0.05).

**Fig. 4:**
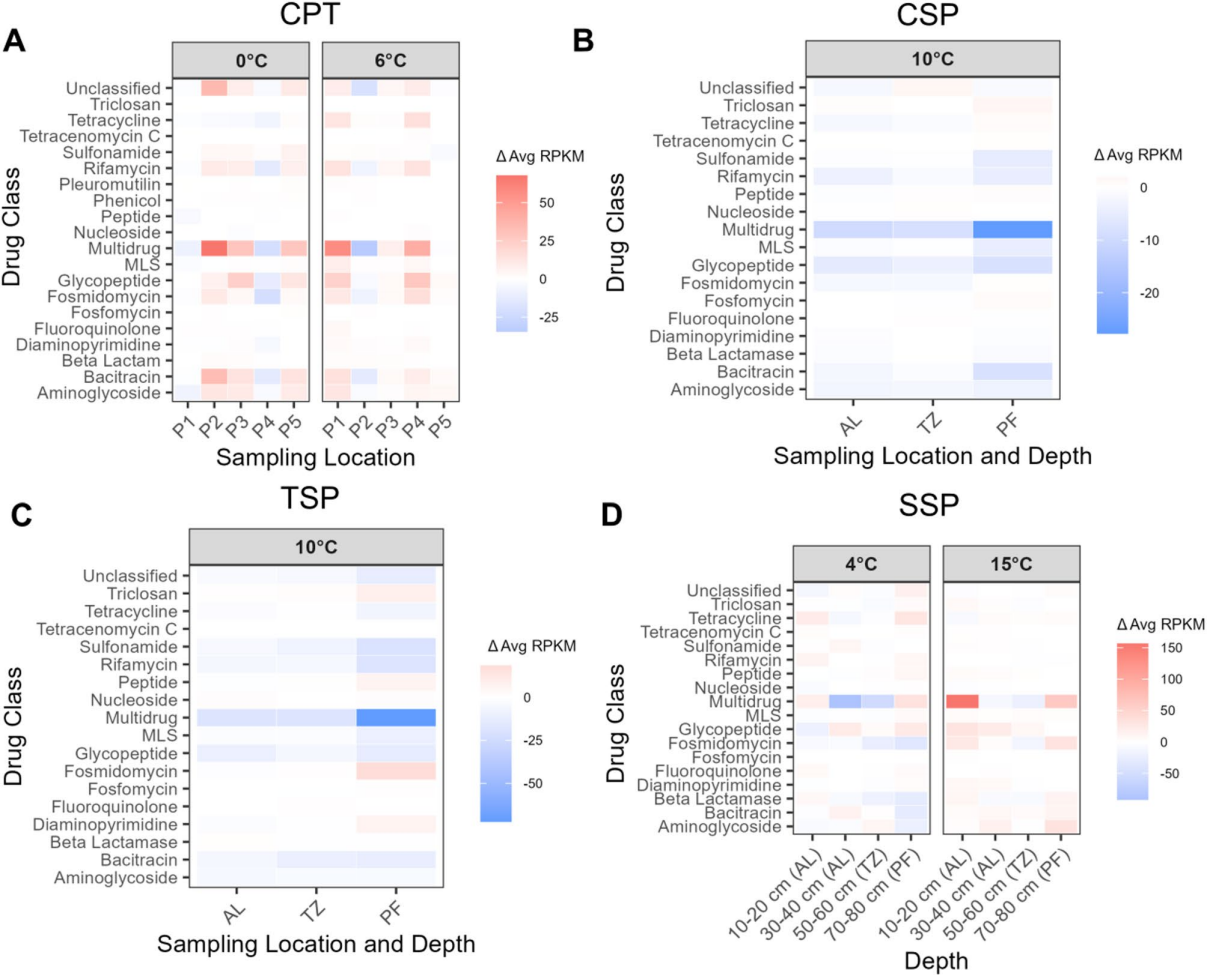
Change in relative abundances of ARG classes following laboratory thaw experiments, as estimated by DeepARG. Data are shown for CPT (**A**), CSP (**C**), TSP (**E**), and SSP (**G**). Values represent change in thawed samples relative to the initial time point. Incubation temperatures are indicated across the top of each panel. Red shading indicates higher relative abundance after thaw, while blue indicates lower abundance. RPKM = Reads Per Kilobase of gene per Million mapped reads. AL= active layer, TZ= transition zone, PF= permafrost.

When comparing abundances of ARGs in each site and soil type before and after incubations, none of the observed changes in ARG class abundance were statistically significant after Benjamini-Hochberg correction (Kruskal-Wallis; *P* > 0.05; Fig. 4, Fig. S9) across the four sites. While differences in several ARG classes were observed in CSP samples (Fig. 4B; adjusted *P* ~ 0.057), these trends were not statistically significant. Both DeepARG and ABRicate showed multidrug resistance classes as potentially decreasing with thaw in the CSP and TSP sites. Patterns of ARG classes varied at differing temperatures in the CPT and SSP datasets. For example, beta lactamase resistance genes decreased in SSP permafrost samples following thaw at 4 °C and increased following thaw at 15 °C (Fig. 4C, Fig. S9C, F) and glycopeptide resistance genes increased at 0 °C in CPT P5, but decreased at 6 °C (Fig. 4A; Fig. S9A).

### Community composition varied across sites and shifted with thaw

**Fig. 5:**
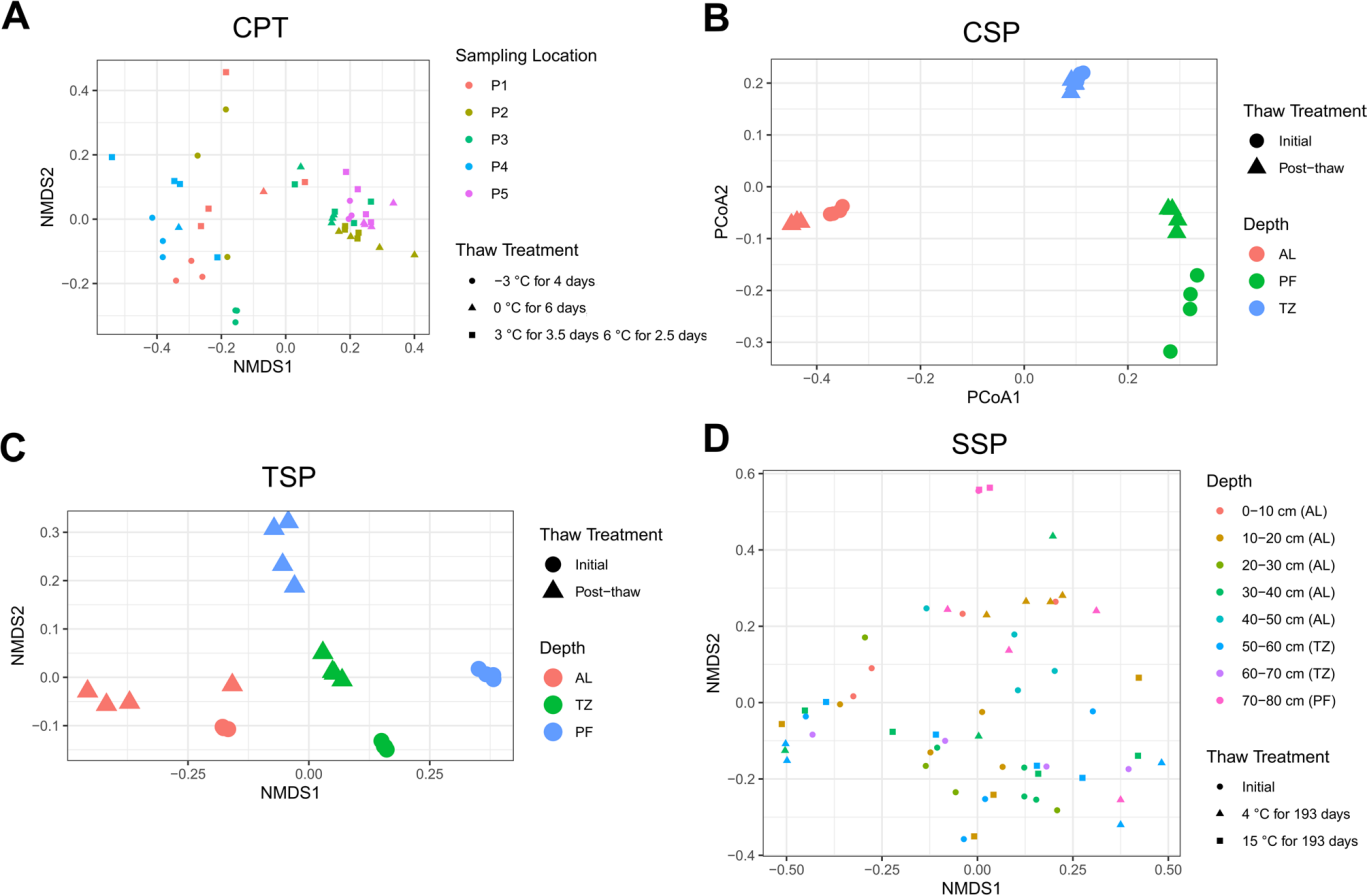
Community composition of shotgun metagenomic reads from laboratory thaw experiments at four permafrost sites. Panels show CPT (NMDS, stress = 0.120; (**A**)), CSP (PCoA; (**B**), TSP (NMDS, stress = 0.126; (**C**)), and SSP (NMDS, stress = 0.190; (**D**)), with taxonomy assigned using MetaPhlAn^56^. Ordinations were calculated on Bray-Curtis dissimilarities. NMDS was used for most sites to represent rank-based dissimilarities, whereas PCoA was used for CSP due to near-zero stress in NMDS resulting from highly dissimilar samples. AL= active layer, TZ= transition zone, PF= permafrost.

To evaluate differences in taxonomic composition between treatments and across sites, we used Bray-Curtis dissimilarity matrices derived from MetaPhlAn profiles that assigned taxonomy to reads (Fig. 5). In the CPT samples (Fig. 5A), microbial community composition differed significantly among the sampling depths at the initial time point (PERMANOVA; R² = 0.73, *p* = 0.001). Community composition and relative abundances of Nitrososphaeria (class) and Thaumarchaeota (phylum) also changed with thaw (R² = 0.15, *p* = 0.003; ANOVA, BH-corrected *P* < 0.05; Fig. S10). For CSP samples, depth had a strong effect on community composition (R² = 0.883, *P* = 0.001) and the relative abundances of classes of Thermolephilia, Actinomycetia, and Acidimicrobiia (Kruskal-Wallis, BH-corrected *P* < 0.05), whereas thaw treatment had no significant effect (R² = 0.001, *P* = 0.881). Similarly, for TSP samples, depth explained a significant portion of variation (R² = 0.550, *P* = 0.001), while thaw treatment was not significant (R² = 0.006, *P* = 0.904). In the SSP site, (Fig. 5C), depth was significant (R² = 0.332, *P* = 0.007) and thaw had a significant effect when all depths were included (R² = 0.054, *P* = 0.038).

Redundancy analysis showed that ARG abundances explained a small and variable portion of microbial community variation across datasets. In CPT, ARG abundance had little effect on variation and did not reach statistical significance (DeepARG: *P* = 0.148; ABRicate: *P* = 0.306;). For CSP samples, DeepARG explained 36.5% (F = 12.66, *P* = 0.001) and ABRicate explained 17.6% (F = 4.69, *P* = 0.003). Similarly, for TSP samples, DeepARG explained 31.0% (F = 9.89, *P* = 0.001) and ABRicate explained 22.2% (F = 6.28, *P* = 0.001). In SSP, the effect depended on the detection method: ARGs quantified with ARGs quantified with DeepARG explained ~ 3.5% of variation (F = 2.04, *P* = 0.015), while ABRicate had a small effect that was not significant.

Analysis of ARGs across the datasets revealed distinct patterns of associations with microbial phyla and classes. In CPT, ARG abundances were strongly positively correlated with relative abundances of Pseudomonadota and specifically within the Alphaproteobacteria class (DeepARG; ρ ~ 0.66–0.68; Pearson; *P* < 0.001; Table S8), indicating a potential link between these groups and the resistome. In CSP, ARGs also exhibited moderate to strong positive correlations with several taxonomic groups, including Actinomycetota, Bacteroidota, Chloroflexota, Actinomycetia, Methanomicrobia, and Sphingobacteriia (ρ ~ 0.41–0.83; Spearman; *P* < 0.001), with multiple taxa detected by both ABRicate and DeepARG. Conversely, negative correlations were observed with Acidobacteriia and Verrucomicrobiota (DeepARG; ρ ~ -0.47 to -0.78; Spearman; *P* < 0.001), suggesting these taxa may be associated with lower ARG abundances. In SSP, ARGs were generally negatively correlated with phyla such as Actinomycetota, Chloroflexota, and Bacteroidota (ρ ~ -0.55 to -0.49; Spearman; *P* < 0.001). However, Euryarchaeota displayed contrasting trends: DeepARG indicated a positive correlation (ρ ~ 0.42; *P* < 0.001).

### Identifying ARGs in metagenome-assembled genomes

Examining high-quality MAGs complements our analysis of ARG abundances across samples by providing a way to link functional potential to specific taxa and identify which microbial populations may harbor resistance traits. Metagenomic assemblies from all four datasets were binned by sample, resulting in ~ 3000 bins that were further refined to high-quality MAGs (> 90% completion and < 5% contamination per CheckM2), yielding 164 MAGs (Table S9). MAGs were assembled from nineteen phyla and represented 105 species (Table S9). Very few MAGs were recovered from the CPT dataset, and Pseudomonadota and Nitrosphaerota were not assembled, despite each representing large portions of reads (Fig. S10). Interestingly, many MAGs assembled from the TSP site belonged to Desulfobacteriota and Patescibacteria, despite their low abundance in the read data. In the SSP dataset, no MAGs were assembled for Euryarchaeota, Candidatus Saccharibacteria, or Gemmatimonadota, whereas Verrucomicrobiota and Patescibacteria were overrepresented.

**Fig. 6:**
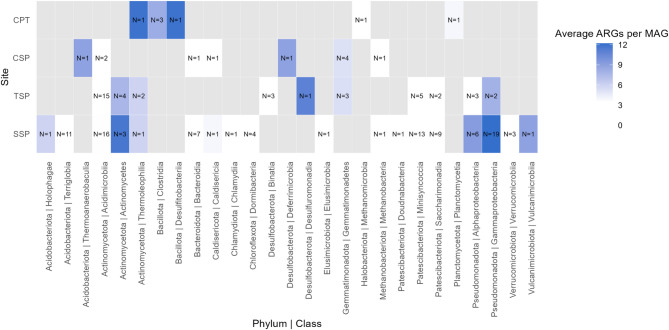
Detection of antimicrobial resistance genes (ARGs) by DeepARG in metagenome-assembled genomes (MAGs) from four permafrost sites. N indicates the number of MAGs in each dataset assigned to the class indicated on the x axis. Grey boxes indicate that no MAGs of that taxonomic group were recovered in the site.

The majority of MAGs contained ARGs, as identified by DeepARG, including 80 out of 105 species. Across the sites, 6 of 7 MAGs in CPT, 10 of 10 MAGs in CSP, 30 of 42 MAGs in TSP, and 79 of 105 MAGs in SSP contained ARGs (Fig. 6; Table S9). MAGs were identified in nine out seventeen phyla and the classes containing MAGs with the most ARGs were Actinomycetes, Thermoleophilia, Desulfidobacteriia, and Gammaproteobacteria (Fig. 6). No ARGs were identified in MAGs belonging to Bacteroidota or Patescibacteria phyla. ABRicate detected few hits in these datasets (Table S9).

## Discussion

Our results reveal variability in the detection, abundance, and distribution of antimicrobial resistance genes (ARGs) across active layer soils and permafrost, with some consistent trends across sites and depths. Differences between ARG detection tools emphasize the importance of considering multiple approaches to capture both high-confidence and potentially novel ARGs. Across sites and depths, ARG abundance and read-based taxonomic assignments varied, reflecting the influence of soil properties on microbial communities. In several sites, intact permafrost contained higher relative abundances of ARGs than seasonally thawed layers. Laboratory thaw incubations ranging from 16 d to 183 d demonstrate stability in relative abundances and class distributions of ARGs, despite shifts in microbial community composition. Together, these findings provide a framework for exploring how ARG detection, environmental context, and thaw shapes the permafrost resistome.

To capture ARGs both closely matching clinical reference databases and more divergent sequences present in environmental datasets, we used ABRicate^43^ and DeepARG^46^ as complementary detection tools. Using default cutoffs, DeepARG identified over 50 times more ARGs than ABRicate across all datasets. In our data, ABRicate detected only a small subset of ARGs, whereas DeepARG revealed a spectrum of resistance, including classes like Bacitracin and Aminoglycosides that were missed by ABRicate (Fig. 4; Fig. S9). ABRicate relies on BLAST-based sequence similarity searches with a 75% identity threshold, limiting detection to known ARGs in reference databases which are largely derived from clinical isolates, so environmental or novel ARGs may be underrepresented^43^ . DeepARG, which has a lower 50% cutoff and is trained on CARD, ARDB, and UniProt databases, can detect ARGs with lower sequence similarity or novel variants, likely explaining the additional classes observed in our samples^46^ . Several ARG classes appear differently between ABRicate and DeepARG due to differences in database classification—for example, polymyxin is categorized as its own class in ABRicate but grouped under ‘peptide’ in DeepARG—rather than reflecting unique detection by one tool over the other.

A previous comparison of ABRicate and DeepARG showed that both tools perform similarly when ARGs are highly abundant; however, in samples with low levels of resistance, such as environmental datasets, DeepARG detects additional true positives and some false positives, that ABRicate does not capture^75^ . Predictions for ARGs with few reference sequences may also be less reliable, since the model has limited examples to learn from^76^ . This can lead to potential overfitting in these underrepresented categories, resulting in precision and recall that vary by antimicrobial class^77^ . Overall, these results highlight the importance of using multiple ARG detection tools. By using ABRicate and DeepARG, we, respectively, captured both the high-confidence ARGs detectable by sequence similarity, and the broader spectrum including low-similarity or potentially novel ARGs. ARGs identified by both tools can be considered robust and highly reliable, whereas those detected only by DeepARG should be interpreted with caution, particularly for classes rarely found in databases, such as Triclosan, Sulfonamide, and Tetracenomycin^46^ .

Prior to incubation, clear differences in both the abundance and taxonomic distribution of ARGs were observed across sites. These sites had distinct differences in biome, permafrost type, and permafrost material (Table S1). Notably, peatland soil (sites TSP and SSP) had overall higher relative abundances of ARGs, which correlated with higher carbon content and lower pH, both of which are characteristic of peatland soil^78^ , as compared to mineral soil (Fig. 2, Table S6). This pattern contrasts findings from other ecosystems, including agricultural soils and Antarctica, where correlations between ARG abundance, carbon, and pH vary widely^22,36,79^ . Such variability suggests that soil carbon and pH alone may not be consistent drivers of ARG abundance; rather, observed relationships may reflect site history, soil type, or other unmeasured environmental factors. Additionally, DNA extraction and library preparation kits have been shown to produce minor differences in taxonomic compositions^80–82^ and introduce GC bias^83^, respectively. Although previous studies indicate that ARG profiles tend to be more stable across different extraction methods^84^, the use of multiple extraction and library preparation protocols across our datasets introduces a possibility that methodological differences contributed to some portion of the observed variation. We do not have standardized controls across extraction methods, so we cannot quantify the magnitude of this effect in our dataset. However, the strong patterns across biome and permafrost material suggest that extraction-related variation alone is unlikely to drive the observed gradients in ARG abundance, but we cannot rule out a larger influence without direct comparisons. Thus, methodological differences remain a potential source of uncertainty in the site-to-site patterns reported here.

Community composition varied across sites and depths in all datasets, yet ARG abundances remained stable (Fig. 2). This decoupling of ARG abundance from overall microbial taxonomy suggests that ARGs may be broadly distributed across multiple taxa, rather than being restricted to dominant or site-specific lineages. This is supported by 76.5% of MAGs from these datasets, representing nine phyla, containing ARGs (Fig. 6), which aligns with findings across numerous soil resistomes identifying diverse hosts of ARGs^31^ . Significant taxonomic changes were observed in most datasets with thaw (Fig. 5), although no clear community shifts were detected in CSP and TSP, likely because these sites were thawed briefly prior to being sampled prior to incubations. Interestingly, ARG abundances decreased in these two sites despite the lack of major community changes, suggesting that factors other than overall taxonomic composition, such as horizontal gene transfer, can influence resistome dynamics during short-term thaw. However, in the CSP site, we can connect the positive correlations between ARG abundances with Actinomycetota and Chloroflexota, while the dataset was shown to have several MAGs belonging to these groups with ARGs (Table S9). This suggests that both taxonomic shifts and potential horizontal gene transfer may contribute to observed ARG patterns. While our results show that ARGs are widespread across permafrost microbiomes, it is important to note that the presence of these genes does not guarantee their expression or confer resistance in situ, which can vary by specific antibiotic class^85^ . Moreover, ARGs may have alternative functions in their native hosts, such as detoxification or signaling^86^ . Nevertheless, the soil DNA pool remains an important reservoir of resistance genes that could be mobilized and expressed by other microorganisms^87^ .

Antimicrobials may be produced when organisms compete for resources^88^ , and production can increase during species interactions^89^ . In two sites, CSP and TSP, ARG abundances decreased after 100 d of incubation, consistently across sites and depths (Fig. 3). In contrast, no statistically significant change in ARG abundance was observed in CPT or SSP samples following thaw, highlighting that responses can vary depending on local conditions. Responses may also vary with incubation time; however, no significant changes were observed in samples with the shortest (CPT; 16 days) and longest thaw times (SSP; 193 days). Additionally, CPT samples were shown to have distinct differences in community composition before and after thaw despite short incubation time. The decrease in ARGs in certain contexts is consistent with observations from Messan et al. ^90^, who reported that antimicrobial metabolite production declined in thawed permafrost, suggesting a potential ecological shift from competition toward more mutualistic or cooperative interactions as permafrost thaws. These patterns may reflect the loss of taxa carrying specific resistance genes or changes in selective pressures that reduce the need for antimicrobial defense.

Our results show that the permafrost resistome remains relatively stable in overall abundance, although resistome dynamics may be influenced by local community composition, environmental conditions, and horizontal gene transfer. Overall, ARG abundance increased with soil carbon content, and at several sites, permafrost contained more ARGs than the seasonally thawed active layer, perhaps due to higher competition under low resource availability in the frozen permafrost environment. As thaw progressed, ARG abundance remained the same at two sites and decreased at two sites, underscoring that resistome responses can differ substantially across permafrost environments. Importantly, we did not observe a consistent increase in ARG abundance with thaw across sites. Looking forward, longer-term incubations, expanded sampling, and transcriptomic analyses will be needed to fully evaluate the potential risks of permafrost thaw for the dissemination of antimicrobial resistance.

## Supplementary Information

Below is the link to the electronic supplementary material.

Supplementary Material 1

Supplementary Material 2

## Data Availability

Raw sequencing reads have been deposited in NCBI GenBank Sequence Read Archive under the following Bioprojects: PRJNA542925 (CPT), PRJNA1173724 (CSP and TSP), and PRJNA657840 (SSP), with accession numbers provided in Table S3. Corresponding metagenome-assembled genomes have also been deposited under the same BioProjects. The code underlying this study is openly available at https://github.com/QLFaber/AMR/.
