# Supplementary material for: Antimicrobial resistance varies with warming in active layer soil and permafrost
Source: Sci Rep. 2026 Apr 14;16:17281. doi: 10.1038/s41598-026-46295-2 (PMC13234164; doi:10.1038/s41598-026-46295-2)
Supplement: Supplementary file 2 — Supplementary Material 2 [file 41598_2026_46295_MOESM2_ESM.docx]

**Supplemental information**

Methods:

*Sample collection and laboratory incubations*

For the CPT dataset, cores were collected in triplicate from 5 different sampling locations within the CRREL Permafrost Tunnel using a SIPRE (Snow, Ice, and Permafrost Research Establishment) corer. Cores were initially stored at -20 °C, transported frozen, then placed at -80 °C. The outside of each core was removed using a microtome blade, then sterile subsamples (~8 g) were extracted from the center of each core in a -10 °C cold room, then samples were incubated in the dark with temperature incrementally increasing over 16 days (-3 °C, 0 °C, 3 °C and 6 °C). The temperature was changed when carbon dioxide efflux rates approached zero, approximately 4 days at -3 °C, 6 days at 0 °C, 3.5 days at 3 °C, and 2.5 days at 6 °C ^1^.

For the Alaska soil profiles, one core was collected to 140 cm at a thermokarst site close to the Toolik Lake Field Station (TSP) and another core was collected to 124 cm at the CRREL Permafrost Tunnel Research Facility in Fox, AK (CSP). Intact cores were collected using a SIPRE corer, then stored at -80 °C for the TSP core and -10 °C for the CSP core. The outside of cores was removed using an ethanol-cleaned microtome blade ^2^. Cores were thawed at 4 °C for 2 days then sectioned by active layer (AL), transition zone (TZ), and permafrost (PF) (Main Text Fig. 1C) each layer was homogenized and stored at 10 °C for 6 days then incubated in the dark for 100 days at 10 °C.

For the SSP dataset, four replicate cores to 80 cm depth were collected using a SIPRE corer and subsectioned into 10 cm sections; thawed active layer was sub-sectioned in the field and the permafrost was cut using sterile handsaws in a 2 °C cold room prior to storage at -20 °C. Small pieces drilled off for the t0 samples and kept frozen prior to DNA extraction. Samples for incubations were thawed for 12 days in transit, then placed at 4 °C for five days prior to incubations. Only the 10-20 cm, 30-40 cm, 50-60 cm, and 70-80 cm sections were incubated. Two subsamples of ~40 g soil for of these depths was placed in specimen cups cleaned with ethanol and incubated in the dark for 193 days at 4 °C and 15 °C.

*Soil properties*

Organic matter content was measured in the CPT samples using the loss on ignition method described by Schulte and Hoskins ^3^. Total carbon (C) and nitrogen (N) were determined in the CSP, TSP, and SSP samples after grinding the soil to a fine powder (CSP and TSP samples were first sieved to 2 mm). Measurements were performed either by dry combustion on a Vario Macro Cube (Elementar, Langenselbold, Germany; CSP and TSP) or via thermal oxidation with gas chromatographic separation and thermal conductivity detection on a Costech C/H/N/S Elemental Analyzer (Costech Analytical Technologies, Inc., Valencia, CA, United States; SSP). Soil pH was measured using site-specific protocols. For the CSP, TSP, and CPT datasets, soil was prepared as a slurry: CSP and TSP soils were combined with deionized water (1:5), shaken for 1 h at 150 rpm, and allowed to settle for 10 min ^4^, while CPT soils were mixed with a 0.1 M calcium chloride solution (1:1) and measured in triplicate at the start of the study. Both datasets were measured using a Hanna Instruments pH probe connected to a SevenEasy S20 pH meter (Mettler Toledo, Columbus, OH, United States). SSP samples were prepared as a fresh soil–water slurry (1:10), shaken for 1 h, and measured using an Accumet basic AB 15 pH meter (Thermo Fisher Scientific, Waltham, MA, United States).


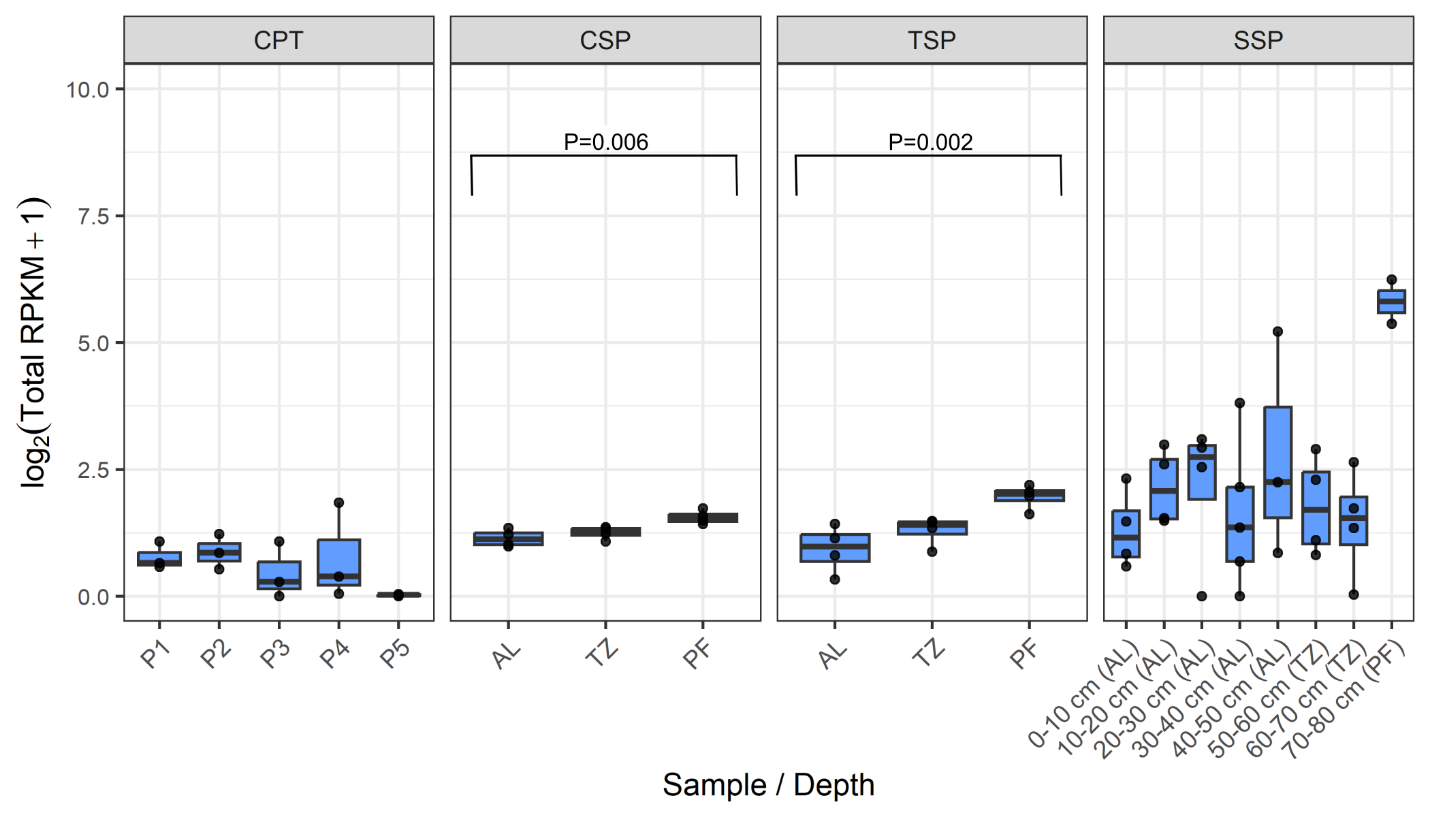


Figure S1. Relative abundance of antimicrobial resistance genes (ARGs) across sample sites prior to incubation, as estimated by ABRicate. RPKM = Reads Per Kilobase of gene per Million mapped reads. RPKM values are displayed as log_2_(RPKM + 1). AL= active layer, TZ= transition zone, PF= permafrost. Error bars indicate standard deviation.


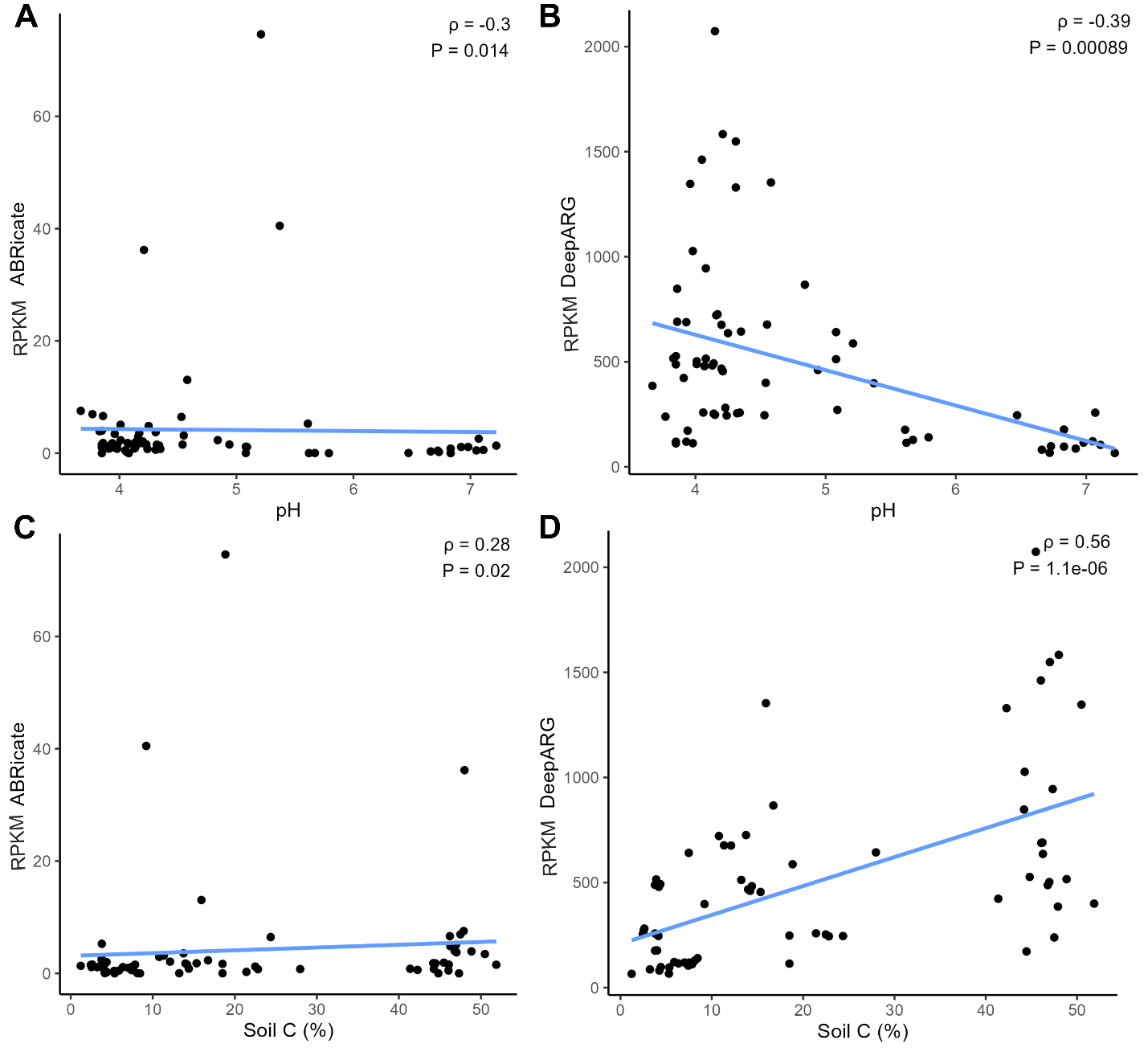


Figure S2. Correlations between soil properties and ARG abundances. A) pH versus ARGs estimated by RPKMs from ABRicate. B) pH versus ARGs estimated by RPKMs from DeepARG. C) Carbon percent versus ARGs estimated by RPKMs from ABRicate. D) Carbon percent versus ARGs estimated by RPKMs from DeepARG. Points represent individual samples; lines indicate Spearman rank correlations. Spearman’s ρ and associated P-values are shown in each panel. RPKM = Reads Per Kilobase of gene per Million mapped reads.


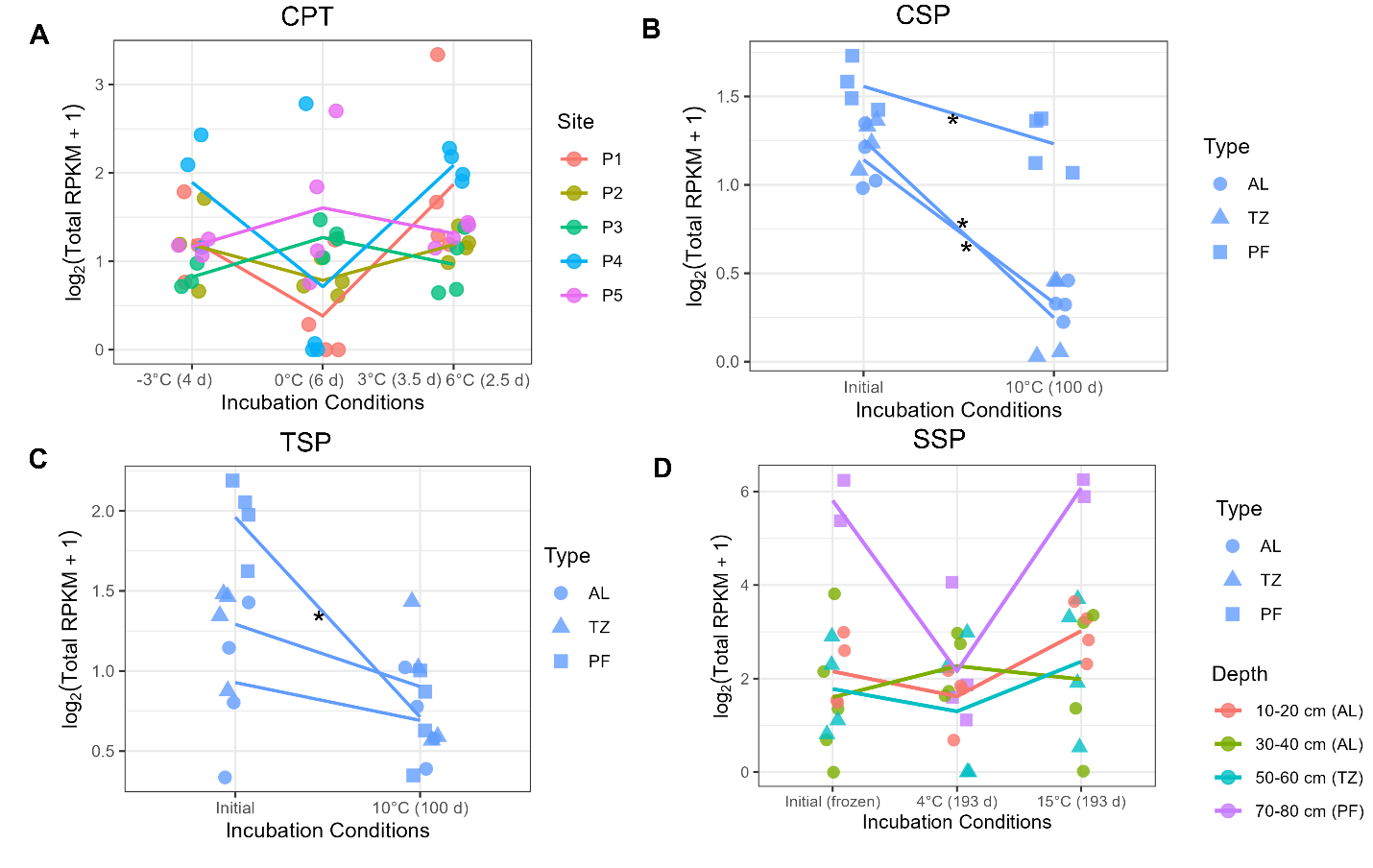


Figure S3. Relative abundance of antimicrobial resistance genes (ARGs) during laboratory thaw experiments, as estimated by ABRicate. Data are shown for the CPT (A), CSP (B), TSP (C), and SSP (D). RPKM = Reads Per Kilobase of gene per Million mapped reads. RPKM values are displayed as log_2_(RPKM + 1). For CPT, CSP, TSP, and SSP, lines connect time points to show changes in ARG abundance. * denotes significant difference (P < 0.05) between pre and post thaw abundances. AL= active layer, TZ= transition zone, PF= permafrost.


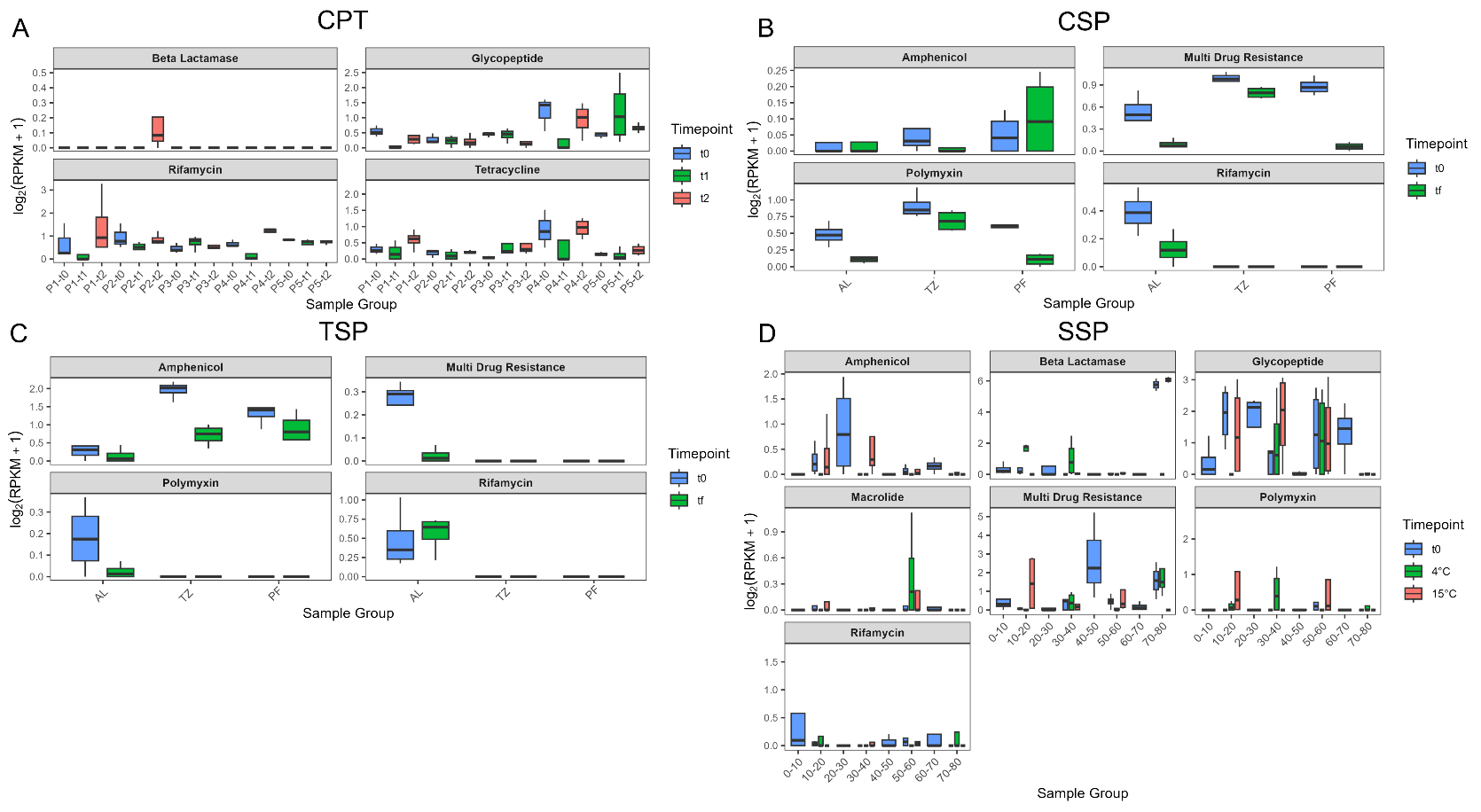


Figure S4. Relative abundances of antimicrobial resistance gene (ARG) classes over laboratory thaw experiments, as estimated by ABRicate. Data are shown for the CPT (A), CSP (B), TSP (C), and SSP (D). RPKM = Reads Per Kilobase of gene per Million mapped reads. RPKM values are displayed as log_2_(RPKM + 1). AL= active layer, TZ= transition zone, PF= permafrost.


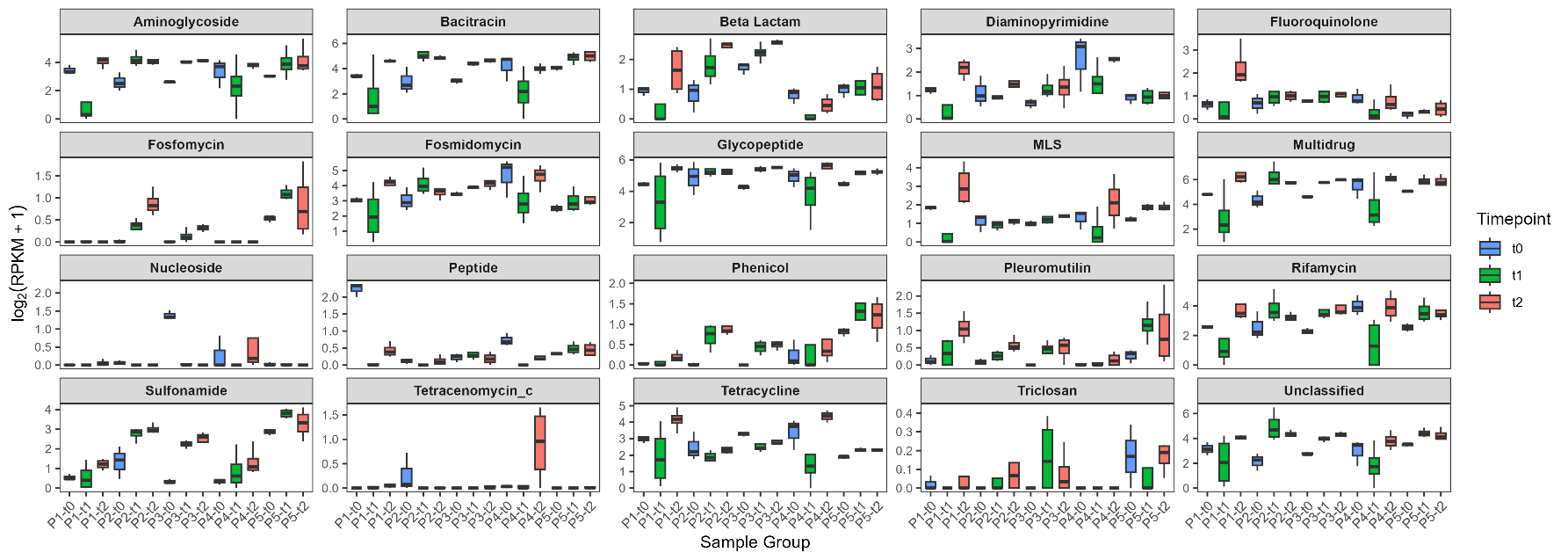
 Figure S5. Relative abundances of antimicrobial resistance gene (ARG) classes over laboratory thaw experiments for CPT, as estimated by DeepARG. RPKM = Reads Per Kilobase of gene per Million mapped reads. RPKM values are displayed as log_2_(RPKM + 1).


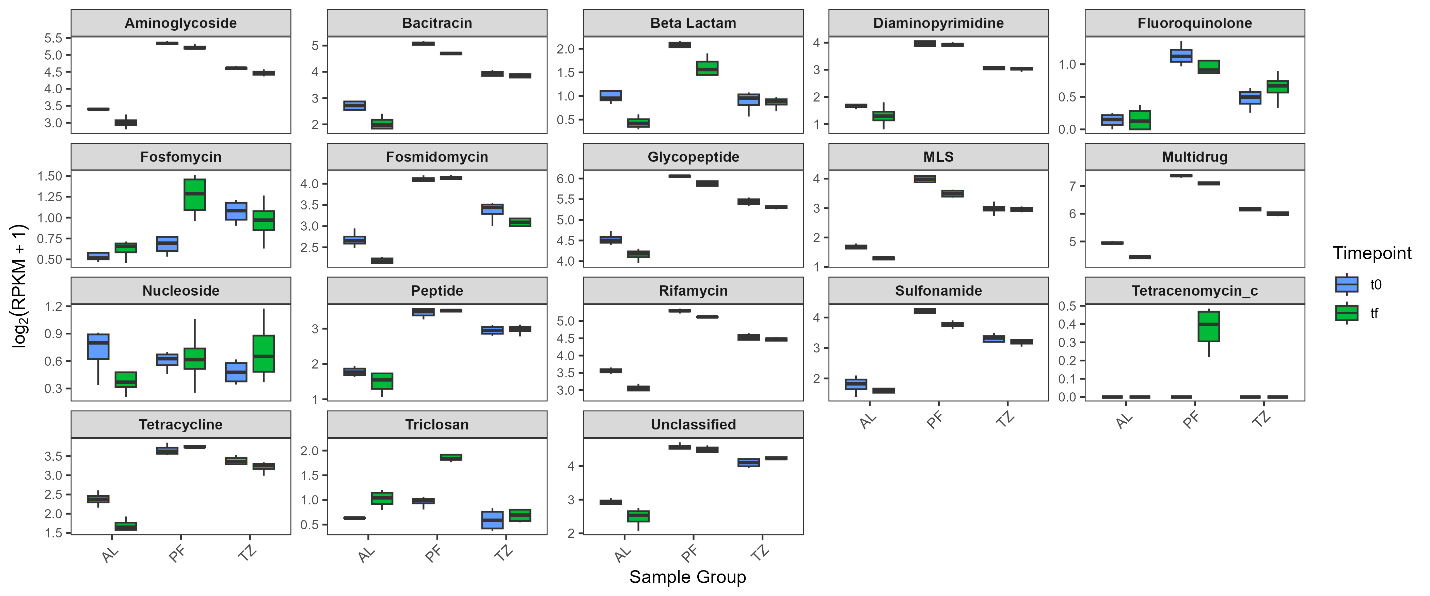


Figure S6. Relative abundances of antimicrobial resistance gene (ARG) classes over laboratory thaw experiments for CSP, as estimated by DeepARG. RPKM = Reads Per Kilobase of gene per Million mapped reads. RPKM values are displayed as log_2_(RPKM + 1). AL= active layer, TZ= transition zone, PF= permafrost.


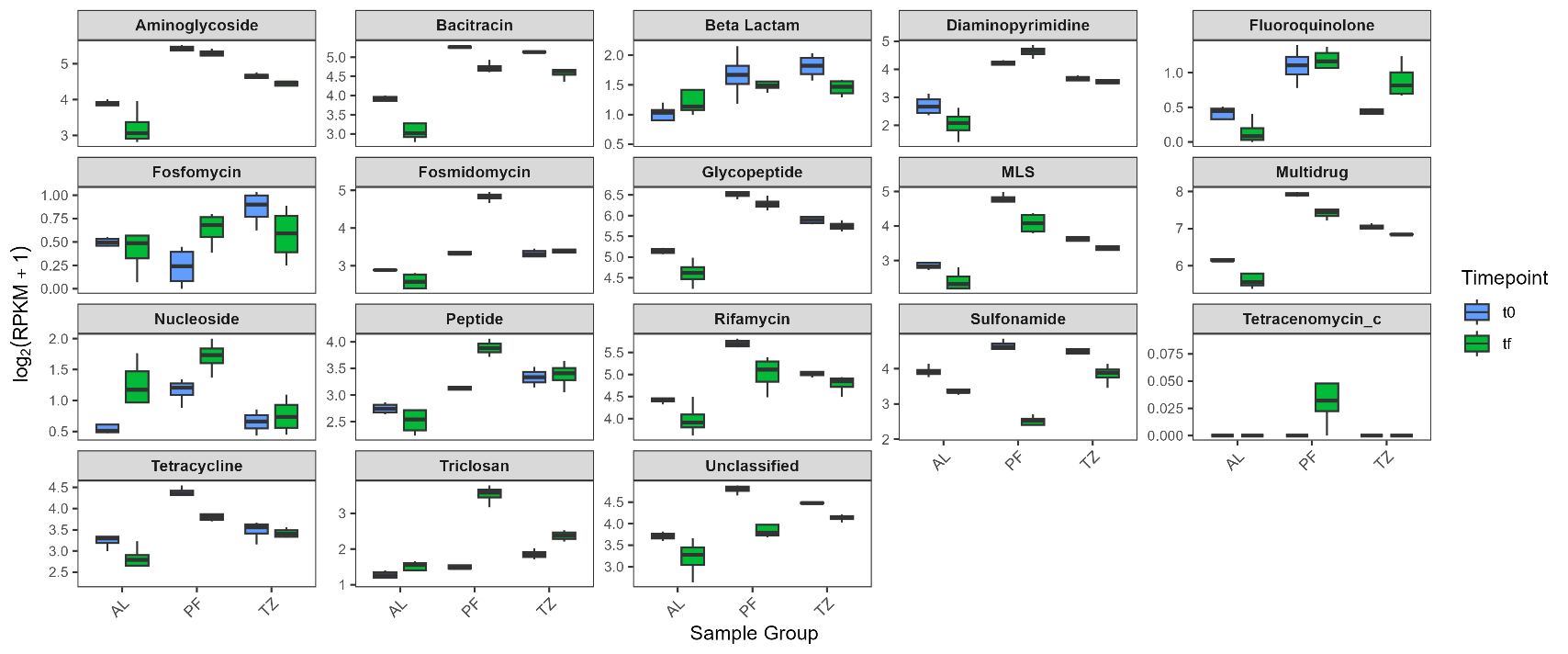


Figure S7. Relative abundances of antimicrobial resistance gene (ARG) classes over laboratory thaw experiments for TSP, as estimated by DeepARG. RPKM = Reads Per Kilobase of gene per Million mapped reads. RPKM values are displayed as log_2_(RPKM + 1). AL= active layer, TZ= transition zone, PF= permafrost.


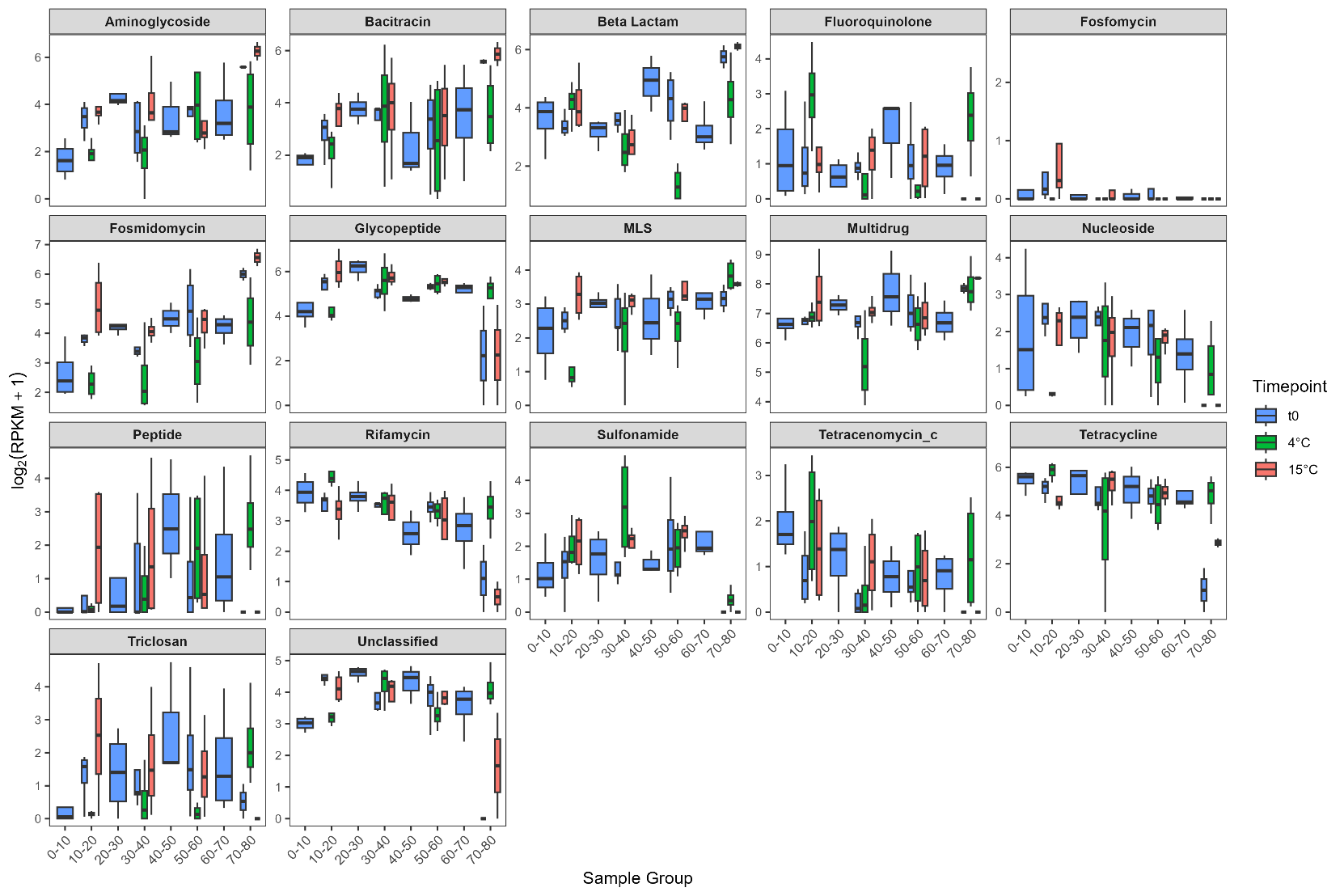


Figure S8. Relative abundances of antimicrobial resistance gene (ARG) classes over laboratory thaw experiments for SSP, as estimated by DeepARG. RPKM = Reads Per Kilobase of gene per Million mapped reads. RPKM values are displayed as log_2_(RPKM + 1).


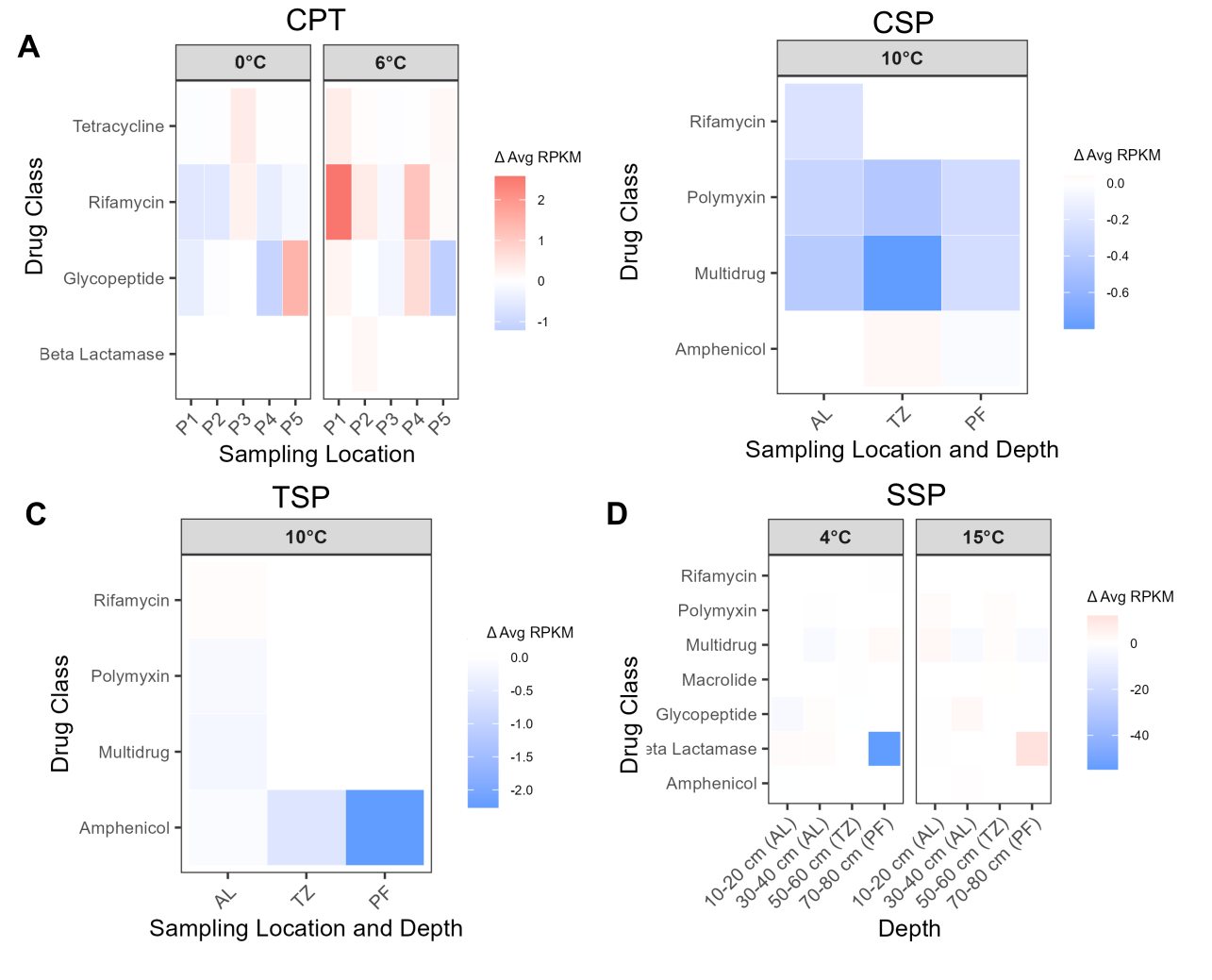


Figure S9. Change in relative abundances of antimicrobial resistance gene (ARG) classes following laboratory thaw experiments, as estimated by ABRicate. Data are shown for the CPT (A), CSP (B), TSP (C), and SSP (D). Values represent change in thawed samples relative to the initial time point. Incubation temperatures are indicated across the top of each panel. Red shading indicates higher relative abundance after thaw, while blue indicates lower abundance. AL= active layer, TZ= transition zone, PF= permafrost.


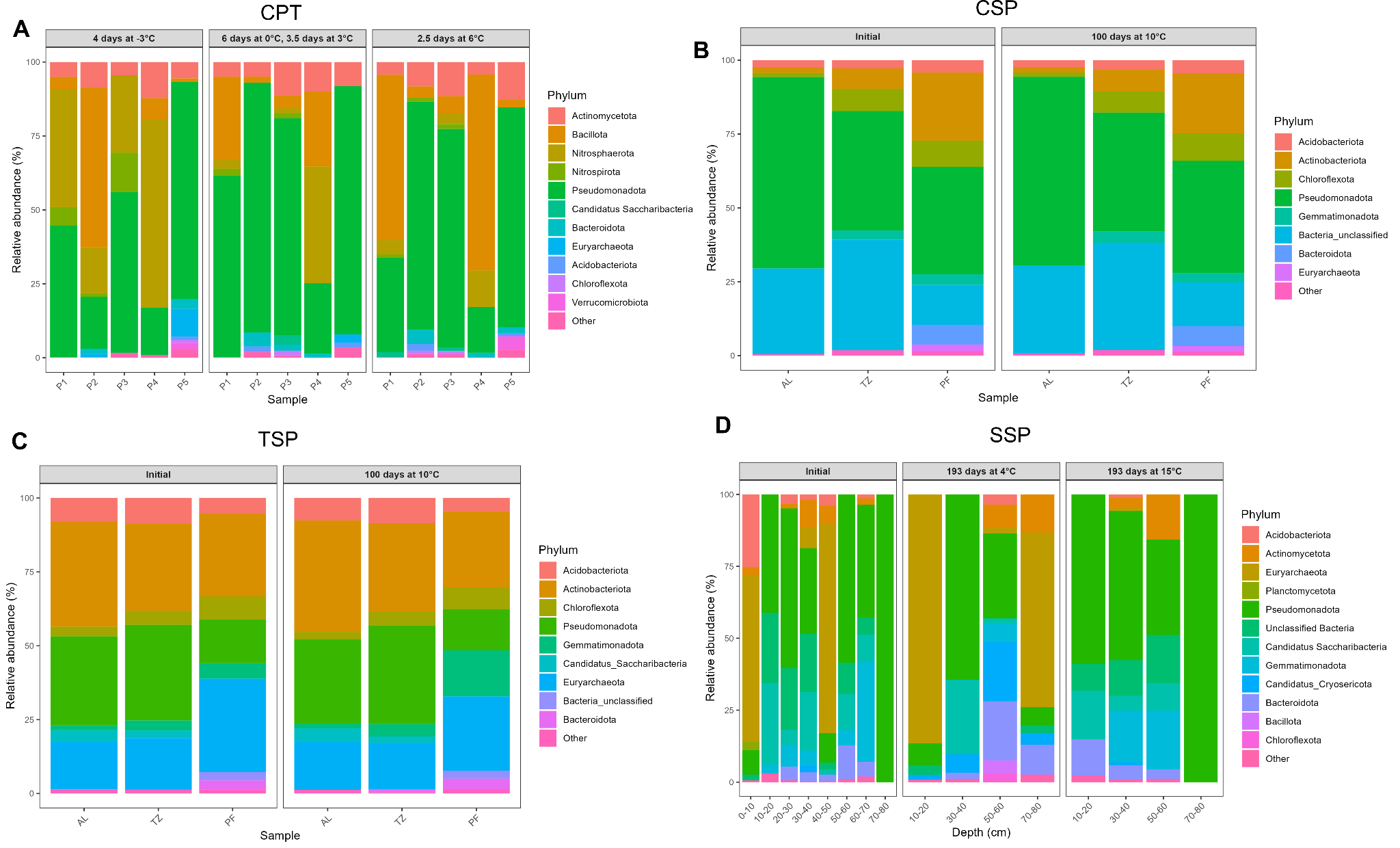


Figure S10. Relative abundance of phyla in laboratory thaw experiments at four permafrost sites. Panels show CPT (A), CSP (B), TSP (C) and SSP (D). Low-abundance phyla (<1%) are combined into Other. Taxonomy was assigned using MetaPhlAn.

Supplemental Tables 1-9: See Excel file.

References

1. Barbato, R. A., Jones, R. M., Douglas, T. A., Doherty, S. J., Messan, K., Foley, K. L., Perkins, E. J., Thurston, A. K., & Garcia-Reyero, N. Not all permafrost microbiomes are created equal: Influence of permafrost thaw on the soil microbiome in a laboratory incubation study in *Soil Biology and Biochemistry* 167 (2022).

2. Barbato, R. A., Garcia-Reyero, N., Foley, K., Jones, R., Courville, Z., Douglas, T., Perkins, E., & Reynolds, C. M. Removal of exogenous materials from the outer portion of frozen cores to investigate the ancient biological communities harbored inside. *Journal of Visualized Experiments* 113 (2016).

3. Schulte, E. E. & Hoskins, B. Recommended soil organic matter tests in *Recommended Soil Testing Procedures for the North Eastern USA* 52–60 (Northeastern Regional Publication, 1995).

4. Kabała, C., Musztyfaga, E., Gałka, B., Łabuńska, D. & Mańczyńska, P. Conversion of Soil pH 1:2.5 KCl and 1:2.5 H2O to 1:5 H2O: Conclusions for Soil Management, Environmental Monitoring, and International Soil Databases. *Pol J Environ Stud* 25, 647–653 (2016).
